# Formal caregivers’ perceptions of quality of care for older people: associating factors

**DOI:** 10.1186/s13104-015-1597-7

**Published:** 2015-10-30

**Authors:** Ingrid From, Bodil Wilde-Larsson, Gun Nordström, Inger Johansson

**Affiliations:** School of Health and Social Studies, Dalarna University, 791 88, Falun, Sweden; Department of Health Sciences, Faculty of Health Science and Technology, Karlstad University, Karlstad, Sweden; Department of Nursing and Mental Health, Department of Nursing, Faculty of Public Health, Hedmark University College, Elverum, Norway; Department of Nursing, Gjövik University College, Gjövik, Norway

## Abstract

**Background:**

Despite the growing number of studies concerning quality of care for older people, there is a lack of studies depicting factors associated with good quality of care from the formal caregivers’ perspective. The aim was to describe formal caregivers’ perceptions of quality of care for older people in the community and explore factors associated with these perceptions. In total, 70 nursing assistants, 163 enrolled nurses and 198 registered nurses from 14 communities in central Sweden participated in the study. They filled out the following questionnaires: a modified version of Quality from the Patient’s Perspective, Creative Climate Questionnaire, Stress of Conscience Questionnaire, items regarding education and competence, Health Index and Sense of coherence questionnaire. The overall response rate was 57 % (n = 431).

**Results:**

In the perceived reality of quality of care respondents assessed the highest mean value in the dimension medical-technical competence and physical technical conditions and lower values in the dimensions; identity-oriented approach, socio-cultural atmosphere and in the context specific dimension. The caregivers estimated their competence and health rather high, had lower average values in sense of coherence and organizational climate and low values in stress of conscience.

**Conclusions:**

The PR of quality of care were estimated higher among NA/ENs compared to RNs. Occupation, organizational climate and stress of conscience were factors associated with quality of care that explained 42 % of the variance. Competence, general health and sense of coherence were not significantly associated to quality of care. The mentioned factors explaining quality of care might be intertwined and showed that formal caregivers’ working conditions are of great importance for quality of care.

## Background

The quality of care for older people is a matter of great interest, since it is an expanding group with multiple illnesses and complex health problems in need of assistance by formal caregivers [1, 2]. Several conceptualizations of quality of care have been developed [3–5]. The present study focuses on the formal caregivers’ perspective when it comes to care for older people. Typically, care in this context involves multiple aspects such as medical and nursing-related, as well as social service related aspects. Given this, a theoretical model of quality of care developed by Wilde et al. [6] was regarded as a suitable point of departure. According to this model, quality of care can be understood as four interrelated dimensions: the caregivers’ medical technical competence, the care organization’s physical-technical conditions, the identity-orientation in caregivers’ attitudes and actions and the socio-cultural atmosphere in the care organization [6]. This model can be regarded as an extension in relation to Donabedian’s basic elements of quality of care which encompass technical care, interpersonal care and amenities [3]. The model [6] correspond also to the quality of care indicators established by the National Board of Health and Welfare [7].

Formal caregivers, such as nursing assistants (NAs), enrolled nurses (ENs) and registered nurses (RNs), are responsible for implementing good quality of care according to legislation and the goals of society. This is an assignment sometimes accessible, other times unachievable [8]. When resources are scarce formal caregivers can feel responsible for shortcomings, although they are not directly responsible for resource allocation [9].

Juthberg [10] illustrated that good care and working conditions, such as organizational climate and stress of conscience, are linked together as the organizational climate affects stress of conscience and vice versa. Hannan et al. [11] also reported that workload, group cohesiveness and commitment were predictors of good quality of care. Moreover, Häggström [8] reported that work overload and lack of time gave formal caregivers feelings of guilt as they had to lower their goals and standards. Prerequisites needed to attain a good organizational climate were that formal caregivers had control over their own work, had fewer working demands and had support from colleagues [12, 13].

Competence has been reported to be important for good working conditions and good quality of care for older people [11]. Pilhammar Andersson [14] has shown that working conditions for formal caregivers improved as a result of education. Lack of competence among formal caregivers could result in a troubled conscience and a feeling of not being able to provide sufficiently good care and was one possible cause of burnout [15].

Formal caregivers’ own health status influenced to a great extent their ability to provide good quality of care [15]. Decreased health status among the formal caregivers could affect the number of errors occurred at work. Formal caregivers who had particularly demanding jobs, physically or emotionally, increased their risk of depression and providing a deteriorated quality of care [16].

Sense of coherence has been shown to be closely related to health, psychosocial conditions and well-being [17]. Individuals who have a strong sense of coherence define events in life as less stressful (comprehensibility). They can mobilize resources in order to deal with stressors (manageability) and have the motivation, desire and commitment to cope in a given situation (meaningfulness) [17]. Formal caregivers who reported that they had a strong sense of coherence and social support at work were protected from problems of ill health [18].

In summary, studies show that many factors can affect the quality of care for older people. Studies have shown that formal caregivers involved in older people’s care may have poor working conditions, lack of competence, and physically and psychologically demanding conditions, which are related to health and coping abilities and have an association to quality of care. These factors have been investigated in studies using bivariate analysis. However, there are a limited number of studies using these factors in multivariate analyses and evidence is still lacking whether the factors above are associated with quality of care. Hence, the aim was to describe formal caregivers’ perceptions of quality of care for older people in the community and explore factors associated with these perceptions.

## Methods

### Setting

In Sweden, community care of older people is funded by each community and is assessed by a care needs assessor. Home care means that care is provided in the person’s own home and can include both medical- and nursing care based on the person’s needs. When older people living in own homes have a greater need for help they are sometimes provided a day-care place receiving care and stimulation during daytime. When having major need of help the older people are often offered the choice of living in a special accommodation where they have staff around the clock [19]. The unit managers in special living accommodations have operational responsibility, responsibility for staff management, social work and collaboration [20]. Registered nurses (RNs) are the highest qualified formal caregivers for health and care in the community and have the responsibility for medical and nursing assessments in direct care, mostly on a consultative basis and for delegation of certain medical tasks to NAs/ENs [20, 21]. They are often responsible for health and care of numerous older people. Accordingly, RNs do not participate in the daily care of the older people in the way that enrolled nurses (ENs) and nurse assistants (NAs) do. The ENs have a nursing education in the upper secondary nursing programme [20]. NAs’ education varies from 10 to 20 weeks of nurse training to studies in the upper secondary nursing programme to no formal health care education at all. The RNs have a three-year academic education in Sweden [22, 23].

### Subjects

In total, 5481 formal caregivers (1767 NAs, 3421 ENs and 293 RNs) employed in 14 communities in one county in Sweden were eligible for this study. In order to obtain distinct appropriate groups approximately 10 %, of NAs (n = 140) and ENs (n = 324) were invited to participate. Due to the limited number of RNs working in these 14 communities all 293 RNs were invited. Of the total 757 formal caregivers who received the questionnaire (see below) the response rate was 57 % (NAs 50 %, ENs 50 %, RNs 68 %). No statistically significant differences were found in a drop-out analysis between respondents and non-respondents in age, time in the occupation, time that they had in their work in older people’s care and time that they had worked in their current workplace.

### Procedure

The executive managers for older people’s care in each of the 14 communities were contacted and gave their permission to contact the employed NAs, ENs and RNs. Subsequently, the unit managers for these formal caregivers were contacted and informed about the study by the first author (IF). Each unit manager informed the group of potential respondents, i.e. the NAs, ENs and RNs, and provided a list to the first author with their names and home addresses. NAs and ENs were selected from these lists by the first author using random numbers.

Each selected participant was sent information to their home address with information regarding the purpose of the study along with a coded questionnaire in a pre-addressed envelope. By returning the completed form, the participant accepted participation in the study. Two reminders were sent out with 2 weeks apart. Data collection was conducted during January to April 2009.

### Measurements

In the present study, a questionnaire including five instruments and demographic items was used.

#### Quality of care

The short version of the Quality from the patient’s perspective questionnaire (QPP) modified for formal caregivers was used [24]. It has 17 items related to the four dimensions in the theoretical model of quality of care [24]: medical-technical competence (6 items), physical technical conditions (2 items), identity-oriented approach (5 items) and socio-cultural atmosphere (4 items). The instrument also contains 9 context-specific items referring to the care of older people in the community. The items were estimated in two ways by the participants: perceived reality (PR) and subjective importance (SI) of quality of care. In the present study, only the PR items were used. An example of an item for PR is “The older people get the best possible help to wash, such as a shower/bath’. Quality of care was valued on a 4-point scale, answering ‘This is what it is like for the older people where I work’”. The response alternatives were from 1 = do not agree at all to 4 = completely agree, and one response 0 = not relevant. The higher the value, the higher the perception of quality of care. The mean values of the items were summed and then divided by the number of items, thus obtaining the average PR total score. This instrument has been used in several national and international studies and has been tested for validity and reliability [24, 25]. In previous studies, internal consistency by means of Cronbach’s alpha for the PR dimensions scales varied between 0.67 and 0.91 [24]. Cronbach’s alpha for the PR total score in the present study was 0.85.

#### Organizational climate

The Creative Climate Questionnaire (CCQ) measures organizational climate [13]. It contains 50 items representing 10 dimensions, which characterize the organizational climate: challenge, freedom, idea support, trust/openness, dynamism/liveliness, playfulness/humor, debates, conflicts, risk taking and idea time. Each dimension contains five items graded on a 4-point scale from 0 = agree not at all to 3 = agree very much. The higher the value, the better the perception of organizational climate, except for conflicts, which is the opposite. The five items in each dimension were added and divided by five to obtain an average value for the dimension. Total mean score was obtained by adding nine of the ten dimensions (all except conflicts) and dividing the sum by nine [13]. In previous studies the values for Cronbach’s alpha for the dimensions varied between 0.66 and 0.90. In this study, Cronbach’s alpha value for the total score was 0.95.

#### Stress of conscience

Stress and the relation to guilt of conscience were estimated using the Stress of Conscience Questionnaire (SCQ) [26]. The questionnaire contains 9 two-part items consisting of an A-part describing a stressful situation on a 6-point scale for the presence of the condition from 0 = never, 1 = less than once/6 months, 2 = more than once/6 months, 3 = every month, 4 = every week and 5 = every day. On the B-part the respondent replies to the degree of guilt for every situation in the A-part, grading bad conscience on a 10 cm visual analogue scale (VAS) answering the question ‘Does this give you a troubled conscience?’ from 0 = no, not at all to 10 = yes, gives me very bad conscience. After dividing the VAS-scores by two, the response scores from A and B respectively were multiplied. The minimum value for an item is 0 and the maximum value 25. Thus the maximum total score per person is 225 [10]. The higher the value, the higher the perceived stress level. Cronbach’s alpha in a previous study for the total score was reported to be 0.83 [26]. In the present study, Cronbach’s alpha was 0.83.

#### Competence

An abbreviated version of the questionnaire on competence development, compiled by Josefsson [22], was used. This questionnaire contains 7 items consisting of statements about whether the formal caregivers’ education had prepared them for their work with response alternatives from 1 = agree not at all, to 5 = agree completely. The higher the value, the higher the estimated competence. The total score was obtained by summing the mean values of all items and dividing by 7. Cronbach’s alpha for the total score in the present study was 0.94.

#### General health

The Health Index (HI) was used to evaluate overall health as it was experienced in the last week [27]. It consists of 10 items, including energy, temper, fatigue, loneliness, sleep, vertigo, bowel function, pain, mobility and general health. The response alternatives varied between 1 = very poor to 4 = very good. The higher the score, the better the perceived self-rated health. The total score ranges between 10 and 40. HI has been found to be a valid and justifiable measure of subjective health with Cronbach’s alpha 0.83 [28]. In the present study the Cronbach’s alpha for the total HI was 0.82.

#### Sense of coherence

The instrument Sense of Coherence (SOC) is intended to measure the ability to manage life based on the dimensions comprehensibility, manageability and meaningfulness [17]. In the present study, the short version with 13 items, rated on a 7-point scale from 1 = never to 7 = very often, was used. The total score ranges from 13 to 91. The higher the score, the higher the sense of coherence. In previous studies Cronbach’s alpha were 0.77 and in [29, 30] the present study 0.83.

### Data analyses and statistics

Demographic data and quality of care were analyzed with descriptive statistics. Pearson’s product-moment correlation was computed between the PR quality of care dimensions. As high correlations were found, the dimensions were merged to a PR total score. Multiple regression analyses were performed with quality of care (PR total score) as the dependent variable. In the initial analysis, the following independent variables were age, gender, occupation, working place, organizational climate, stress of conscience, competence, own health and sense of coherence. The variables were entered into the model simultaneously (‘enter’).

For the final analysis, all the significant independent variables, occupation, organizational climate and stress of conscience, from the initial model were included. No multicollinearity was present. Analyses were performed using SPSS version 18.0. A *p* value <0.05 for statistical significance was used.

### Ethical considerations

After obtaining the names of the chosen participants, they received an information letter which was attached to the questionnaire informing them about the study and that their participation was voluntary. The participants gave written informed consent in connection with their response to the questionnaire. All participants were guaranteed confidentiality. The study was reviewed by the local committee for research ethics at Karlstad University (Dnr C2008/537), which gave permission to conduct the study.

## Results

### Formal caregivers’ characteristics and perception of their personal and work related variables

Table 1 shows characteristics of the sample and mean values of investigated variables. The formal caregivers were average 48.2 years old and only a few were men. Most of the formal caregivers worked in institutions for older people (Table 1). They had worked in the current workplace for 10.2 years and in older people’s care for a mean 18.3 years (not shown in table). Of all the formal caregivers 7 % (n = 28) had no formal health care education (not shown in table).

**Table 1:** Formal caregivers characteristics and perceptions of personal and work related variables

	Total, n = 431	
Age years M (SD)	48.2 (10.5)	
Years in current work place: M (SD)	10.2 (8.5)	
Gender, n (%)		
Women	411 (95)	
Men	20 (5)	
Work place, n (%)		
Home help service	63 (15)	
Institutions for older people	336 (80)	
Home help service and institutions	21 (5)	
Working hours, n (%)		
>75 %	244 (57)	
≤75 %	183 (43)	

The caregivers’ perception of competence, health, sense of coherence, stress of conscience and organizational climate are shown in Table 2. The caregivers assessed in average their competence as rather high 3.75 (SD = 1.06) and the estimation of their health showed relatively high values 31.59 (SD = 3.99). Lower average values were seen in sense of coherence 58.79 (SD = 5.63) and in organizational climate 1.63 (SD = .52). Stress of conscience was (valued low); in average 37.24 (SD = 31.21), indicating low level of stress of conscience.

**Table 2:** Caregivers perception of competence, health, sense of coherence, stress of conscience and organizational climate

Total scores	M	(SD)
Competence^a^	3.75	(1.06)
Health^b^	31.59	(3.99)
Sense of coherence^c^	58.79	(5.63)
Stress of conscience^d^	37.24	(31.21)
Organizational climate^e^	1.63	(0.52)

^a^Answers from 1 = not at all to 5 = agree completely and 0 = not relevant. A high value means to be better prepared

^b^Answers ranging between 10 and 40. A higher score a better health

^c^Answers ranging between 13 and 91. A higher score a higher sense of coherence

^d^Answers ranging between 0 and 225. The higher the more stress of conscience

^e^Answers from 0 = agree not at all to 3 = agree very much. The higher mean values the better climate

### Quality of care

In quality of care the total mean value of PR was 2.9 (SD = 0.47). Table 3 shows that in PR quality of care the highest mean values were found in the dimension medical-technical competence (M = 3.3; SD = 0.64) and physical technical conditions (M = 3.2; SD = 0.55). Lower values were found in the dimensions identity-oriented approach (M = 2.7; SD = 0.63), socio-cultural atmosphere (M = 2.7; SD = 0.64) and in the context specific dimension (M = 2.8; SD = 0.50).

**Table 3:** Caregivers perception of perceived reality of quality of care in community care

PR^a^	X	(SD)
Total	2.9	(0.47)
Medical-technical competence	3.3	(0.64)
The older people receives best possible help with their hygiene, as for example shower/bath	3.47	(0.71)
The older people receives best possible help with dental care	3.02	(0.86)
The older people receives best possible help with hair care	3.25	(0.79)
The older people receives best possible help with toileting	3.44	(0.72)
The older people receives best possible help to sit and lie down comfortably	3.39	(0.70)
The older people receives best possible help with meals	3.38	(0.71)
Physical technical conditions	3.2	(0.55)
The older people receive food and drink that they like	2.70	(0.92)
The older people have access to the help aid they need, such as a wheelchair, walker, etc.	3.75	(0.50)
Identity-oriented approach	2.7	(0.63)
The caregivers seem to understand how the older people experience their situation	2.87	(0.75)
The caregivers meet the older people with respect	3.13	(0.76)
The caregivers show commitment; “show that they care about the older people”	3.16	(0.76)
The older people get good information about changes in help; for example who and when somebody is coming	1.91	(1.16)
The older people have good opportunities to participate in the nursing care	2.25	(1.03)
Socio-cultural atmosphere	2.7	(0.64)
The older people have the opportunity to do the things they wish to do	1.97	(0.97)
The older peoples’ care are determined by their own requests and needs rather than the caregivers’ procedures	2.45	(0.94)
The older peoples’ relatives are treated well	3.35	(0.77)
There is a pleasant atmosphere on the ward	2.85	(0.91)
Context specific	2.8	(0.50)
The older people get best possible help with training	2.24	(0.94)
The older people get best possible help to get outdoors	1.92	(1.00)
The caregivers have a soft and gentle touch	3.03	(0.75)
The caregivers have time for the help they carry out- they do not have to rush	2.33	(0.86)
The older people get help from people they recognize	2.88	(0.84)
The older people have the opportunity to sometimes wish special food	1.66	(1.21)
The older people have a room of their own	3.56	(0.87)
The older people have room enough to have their own personal belongings that are important to them	3.56	(0.88)
The older people have possibilities to receive visits from their relatives in their accommodation	3.70	(0.74)

^a^Perceived reality (PR) scale; 1 = do not agree at all to 4 = fully agree

### Factors associated with quality of care

The initial model in the multiple regression showed that three independent variables (occupation, organizational climate and stress of conscience) were significantly associated with quality of care (the dependent variable) (Table 4).

**Table 4:** Multiple regression analysis of factors potentially influencing perceived reality of quality of care

Independent variable	B	Std err	t	p-value
Initial model				
Gender				
Female	0.141	0.091	1.554	0.121
Male	0			
Occupation				
NA/EN	0.299	0.044	6.750	0.000
RN^a^	0			
Work place				
Home help service	−0.006	0.121	−0.047	0.963
Institutions for older people	0.156	0.107	1.469	0.143
Both in home help service and institutions^a^	0			
Time at current work place	0.000	0.002	0.110	0.913
Organizational climate	0.440	0.045	9.871	0.000
Stress of conscience	−0.003	0.001	−3.814	0.000
Competence	0.004	0.003	1.271	0.205
Sense of coherence	0.003	0.004	0.630	0.529
Health Index total	−0.001	0.006	−0.152	0.879
Initial model: R squared = 0.453, adjusted R squared = 0.433				
Final model				
NA/EN	0.254	0.040	6.307	0.000
RN	0			
Organizational climate	0.414	0.040	10.238	0.000
Stress of conscience	−0.003	0.001	−4.472	0.000
Final model: R squared = 0.422, adjusted R squared = 0.417				

^a^These parameters are set to zero because they are redundant

The final model (Table 4) showed that the independent variables occupation, organizational climate and stress of conscience together explained a total of 42 % of the variance. NAs/ENs estimated quality of care higher than the RNs. Moreover, the better organizational climate, the higher the quality of care. Mean difference in organizational climate could be 1.2 (minimum = 0, maximum = 3). For stress of conscience in relation to quality of care there was a negative association, namely, the more stress of conscience, the lower the quality of care and vice versa. The highest mean difference for stress of conscience could be −0.477 (minimum = 0, maximum = 158.95).

## Discussion

The result showed that the caregivers’ perception of their competence and health were estimated relatively high, and lower values were found for sense of coherence and organizational climate. Stress of conscience was also estimated low by caregivers which means low stress of conscience levels. The highest value of caregiver’s perceived reality of quality was seen in the dimensions medical-technical competence and physical technical conditions. The lowest values were seen in the identity-oriented approach, socio-cultural atmosphere and in the context specific dimension.

Factors that were associated with formal caregivers perceived quality of care was occupation, organizational climate and stress of conscience, together explaining 42 % of the variance. However, the independent variables gender, competence, the formal caregivers’ own health and sense of coherence were not significantly associated with quality of care, although significant differences have been reported in earlier studies using univariate methods [11, 15, 31].

One factor that was associated with perception of quality of care was the formal caregivers’ occupation, whereas the NAs/ENs assessed quality of care higher than the RNs. Several other studies have shown that formal caregivers (NA/ENs, RNs) were critical with regards to quality of care [9, 10, 19, 32, 33]. In these studies different explanations of the difference between the NA/ENs and RNs assessment of quality of care were given. Wreder [33] argued that it might be easier for the RNs to criticize the quality of care since they became distant viewers of the care performed by the NA/ENs, while Juthberg [10] stated that RNs were critical to the quality of care they themselves provided. A problem that may arise due to the RNs’ larger working area in older people’s care, could be inadequate control and problems with meeting the commitments embodied in their professional role. According to Karlsson et al. [32] the RNs were aware of the fact that there were expectations on themselves as being able to provide nursing care on a specialist level without having specialist education. The RNs could experience themselves as being inadequate, incompetent and powerless [10]. Further Karlsson et al. [32] described that RNs in their professional role had to ‘be everywhere and to know everything’ [32, p. 265] and yet they perceived that they were not appreciated for their work. Receiving appreciation and confirmation in the professional role has been reported to be important not only for professional development but also for quality of care [34]. However in the study by Juthberg [10] also NAs reported that they were in a vulnerable position and could be prevented from being good caregivers because of the organization, for example heavy workload and regulations.

Organizational climate was significantly associated with quality of care in the present study, whereas better organizational climate resulted in higher values of quality of care. This result is supported by other authors [13, 35]. According to Ekvall [13], individuals working in a creative organizational climate, in contrast to a stagnant climate, experience challenge at work, are motivated, enjoy work and find fulfillment in their work. They have freedom to carry out their work independently, new ideas are met positively and they have secure relationships. There is also an easy atmosphere, with a strong dynamism in the organization stimulating the innovative capacity of individuals and increasing the quality of their work. Mattiasson [36] showed in a study among formal caregivers in nursing homes that organizational climate was associated with patient autonomy because formal caregivers were more permissive and allowed the older people to decide for themselves to a greater extent. In Sweden, studies have been carried out in the organization of care of older people in other communities similar to these communities in the present study, and showed that the RNs had difficulties meeting their own professional standards [9, 34]. Further, studies have shown that formal caregivers had short-term solutions of complex caring and nursing problems with low quality of care [9, 35, 37]. Since NAs, ENs and RNs in Sweden do not always work together on a daily basis, there might be problems with setting aside appropriate time for developing a good atmosphere and trusting relationships, which according to Ekvall [13] are the basis in good cooperation, exchanging of ideas and quality of care.

In our study, stress of conscience was significantly associated with quality of care. Higher stress of conscience was associated with lower levels of perceived quality of care. In a study by Häggström [9] a deteriorating quality of care caused by stress of conscience had its origins in feelings of being let down by their employer because of an overwhelming workload. Further the formal caregivers described feelings of guilt and betrayal of the older people by not giving them enough attention, being inadequate in their work, ignoring older people’s pleas for help, and only having time for the most basic needs [9]. Juthberg [10] reported that formal caregivers who managed a balance between external expectations and internal demands could maintain their identity of being good caregivers. The result from the present study showed that the formal caregivers’ perceptions of quality of care were related to working conditions such as organizational climate and stress of conscience. This is supported by other studies showing that stress of conscience was a result of bad working conditions and deteriorating quality of care [10, 26].

### Methodological considerations

In the present study, the five instruments used and the seven items connected to education and competence have been tested for reliability by means of Cronbach’s alpha. The values for the instruments and the seven items indicated good internal consistency as they varied between .67 and .94, with exception for the PR dimension physical-technical conditions of quality of care which was low and therefore the items were treated separately [38].

One advantage in this study is the randomized selection of the NAs/ENs. The response rate was 57 % in total, among the NAs and ENs 50 %, while 68 % of the RNs responded. The random selection of the NAs/ENs, is a strength, since a loss of respondents will be distributed in such way that the groups are likely to still have the same representation in the population. Since the selection plan was carefully considered the intended population may still be representative. There were no significant differences between respondents and non-respondents in relation to age, time in occupation, time working in older people’s care and time working in their current workplace.

The response rate (57 %) in this study is regarded as acceptable for a questionnaire survey (38). It is a strength that the rate of internal drop-outs were found to be low or moderate for all the questionnaires except for the SCQ.

Results from qualitative studies about older people’s perception of good care [39] confirmed almost the same topics as were investigated among the formal caregivers in the present study (e.g. safe and secure care, time and continuity). When comparing QPP in in studies with different populations higher values were found among patients in older people’s care [40] and patients in emergency hospital care [41]. Lower values were shown in adults with developmental disabilities [42] among their caregivers and relatives [40, 43], among family members who are informal caregivers and family members of nursing home residents [44]. The three variables explained 42 % of the formal caregivers’ perceptions of quality of care. This indicates that the formal caregivers’ views on quality of care include additional factors which we did not manage to capture.

### Conclusions and implications for practice and future research

The caregivers in this study assessed quality of care rather high. The highest average value were found in the dimension medical-technical competence and physical technical conditions and lower values in the other quality of care dimensions. Their competence and health were valued relatively high by the caregivers, but lower values for sense of coherence and organizational climate were shown. Low levels of stress of conscience were reported.

Occupation, organizational climate and stress of conscience together explained 42 % of the variance of the formal caregivers’ perception of quality of care. More studies are needed to explain additional factors of quality of care as for example social and psychological factors.

This findings showed that the formal caregivers rated quality of care differently i.e. the NA/ENs assessed quality of care higher than RNs, which could affect the performance of the work and therefore need to be studied further.

Organizational climate and stress of conscience seems to be intertwined and were associated with formal caregivers’ perceptions of quality of care. This result motivates the need of improvement in this area in order to develop quality of care for older people.
